# Recent Increasing Incidence of Early-Stage Cervical Cancers of the Squamous Cell Carcinoma Subtype among Young Women

**DOI:** 10.3390/ijerph17207401

**Published:** 2020-10-12

**Authors:** Takafumi Noguchi, Masayoshi Zaitsu, Izumi Oki, Yasuo Haruyama, Keiko Nishida, Koji Uchiyama, Toshimi Sairenchi, Gen Kobashi

**Affiliations:** 1 Department of Public Health, School of Medicine, Dokkyo Medical University, Mibu, Tochigi 321-0293, Japan; m-zaitsu@dokkyomed.ac.jp (M.Z.); yasuo-h@dokkyomed.ac.jp (Y.H.); tossair@dokkyomed.ac.jp (T.S.); genkoba@dokkyomed.ac.jp (G.K.); 2 Department of Adult Nursing, Dokkyo Medical University School of Nursing, Mibu, Tochigi 321-0293, Japan; 3 Division of Cancer Information and Prevention, Tochigi Cancer Center, Utsunomiya, Tochigi 320-0834, Japan; i-oki@tochigi-cc.jp; 4 Department of Obstetrics and Gynecology, Faculty of Medicine, University of Tsukuba, Tsukuba, Ibaraki 305-8575, Japan; keikonsd@dokkyomed.ac.jp; 5 Laboratory of International Environmental Health, Center for International Cooperation, Dokkyo Medical University, Mibu, Tochigi 321-0293, Japan; koji-u@dokkyomed.ac.jp

**Keywords:** cervical cancer, incidence, screening, squamous cell carcinoma, histology subtype

## Abstract

Few studies have reported on the increase in cervical cancer incidence in Japan. We aimed to determine the relevant trends in the metropolitan regions of Japan and to identify the population with the highest risk, based on histological subtype, cancer stage, and diagnostic processes. Using population-based data (2009–2013), we identified 2110 women, aged ≥20 years, with cervical cancer. We estimated the age-standardized and age-specific incidence rates of cervical cancer for the study period based on the 1985 national model population. The average annual percent change (AAPC) and 95% confidence interval (CI) were calculated using the joinpoint regression analysis. We stratified the analyses based on histological subtypes, stage, and diagnostic process via cancer screening. The increase in the overall age-standardized incidence was not significant. However, the increase was significant for women aged 30–39 years (AAPC 20.0%/year, 95% CI: 9.9–31.1), which was attributable to the increase in the incidence of the squamous cell carcinoma (SCC) subtype (AAPC 23.1%/year, 95% CI: 10.7–36.8). Among younger women, aged <50 years, further stratification showed an increase in the undiagnosed early-stage SCC subtype via cancer screening. In Japan, the incidence of HPV-related cervical cancer has been increasing in undiagnosed younger women.

## 1. Introduction

Cervical cancer is the fourth most common cancer that affects women worldwide, in addition to ranking fourth among the causes of cancer-related mortality [1]. In Japan, the estimated number of patients with cervical cancer newly diagnosed in 2016 was approximately 34,000 (23,000 cases of carcinoma in situ (CIS) and 11,000 cases of invasive cancer) [2]. The incidence of cervical cancer remains higher in developing countries than in developed countries [1]. For instance, in 2013, the age-standardized incidence rates for invasive cervical cancer, defined by the diagnosis code C53 in the International Classification of Diseases, 10th revision (ICD-10), were 15.7/100,000 population in developing countries and 9.6/100,000 population in developed countries [3]. Nevertheless, the global trend in terms of incidence has been declining as a result of the implementation of two critical prevention strategies: human papillomavirus (HPV) vaccination and early detection via cancer screening (Papanicolaou (Pap) smear and HPV test) [1,2,3,4].

Compared with other developed countries, some urgent concerns have been raised in Japan regarding the prevention of cervical cancer. In 2013, the Japanese government suspended proactive recommendations for HPV vaccination owing to suspected adverse events, such as complex regional pain syndrome, which resulted in low HPV vaccination rates (<1% to date) [4,5]. The cervical cancer screening rate is lower in Japan (42.4%) than in other Western countries (83.3% in the United States and 80.0% in Italy) [6].

Furthermore, the high ratio (23,000/11,000 cases) for newly diagnosed patients with CIS and invasive cervical cancer may also reflect the failure of cervical cancer prevention in Japan [2]. Indeed, a few recent studies have highlighted an increase in the incidence of cervical cancer during the last two decades in this country, contradicting the current global trend [7,8,9]. For instance, a significant average annual percent change (AAPC) of 1.2% (95% confidence interval (CI): 0.2–2.2) was observed in the incidence of invasive cervical cancer from 1991 to 2010 [7], and a substantial increase (AAPC 17.9%, 95% CI: 10.5–25.8) was also observed in the incidence of CIS from 2006 to 2012 [8]. A continuous increase in the incidence of squamous cell carcinoma (SCC) cervical cancer has been observed in Osaka prefecture since 2000 (although this study included cancer of the corpus uteri) [9]. However, in another study, the incidence of the SCC subtype, the most common (~90%) HPV-related histological subtype of cervical cancer, was null or showed a potentially decreasing trend (AAPC −0.4%, 95% CI: −2.2 to 1.5) [8]; thus, there is ambiguity regarding the actual trend. Therefore, we aimed to further highlight and investigate this unexpected increase in cervical cancer incidence in this high-risk population for which the national prevention strategy has achieved much less than the level for which there is a global consensus. Additionally, to the best of our knowledge, the increase in cervical cancer incidence has not been fully assessed in terms of age, histological subtypes, cancer stage, and diagnostic processes [10,11,12,13].

In this study, we examined the trends of cervical cancer incidence in Japan. Using a population-based data set with >2000 patients with cervical cancer in Tochigi prefecture, we sought to determine whether the incidence of cervical cancer was increasing. Furthermore, we sought to determine whether the increasing trend, if any, differs in terms of age, histological subtypes, cancer stage, and diagnostic processes.

## 2. Materials and Methods

### 2.1. Data Sources and Study Subjects

We obtained a population-based dataset of patients, aged ≥20 years, with cervical cancer, registered in the Tochigi Cancer Registry (TCR) from 2009 to 2013. Tochigi prefecture, which has a population of nearly 2 million (approximately 1.5% of the Japanese population), is located approximately 100 km north of Tokyo. The major industries in Tochigi prefecture are related to manufacturing and agriculture/forestry [14]. The obtained dataset included basic information on patients with cervical cancer (age and date of diagnosis), clinical information (diagnosis, pathology, and stage), and information regarding diagnostic processes. Because of limited data accessibility, survival data were not available. The Tochigi model population data were obtained from the National Cancer Center [2].

We identified 2170 patients registered in the TCR with a diagnosis of cervical cancer (C53 and D06 in ICD-10) [8]. We then excluded patients for whom information on stage (15 patients, 0.6%) and diagnosis process (45 patients, 2.0%) was not available. A total of 2110 patients with cervical cancer having complete data were analyzed. In this study, the death certificate only (DCO) was 0.6% during the study period; hence, the quality of this registry dataset was deemed appropriate for analysis in accordance with a previous study [15]. The Tochigi prefecture and the Bioethics Committee of Dokkyo Medical University approved this study (Protocol No. 29006).

### 2.2. Definition of Histology, Cancer Stage, and Diagnostic Process

According to previous studies [8], we classified histological subtypes (identified according to the International Classification of Disease for Oncology, Third Edition pathological codes) into SCC (8051–8084 and 8120–8131), adenocarcinoma (8140–8490), and other subtypes (8000–8045 and 8560–8900). It should be noted that, in this study, severe dysplasia (cervical intraepithelial neoplasia 3) was included in CIS. We classified cancer stages, based on the surveillance, epidemiology, and end results system, into four categories (CIS, localized, regional, and distant metastasis) [9,16]. According to the International Federation of Gynecology and Obstetrics (FIGO) classification, stages IA–IB2, IIA–IVA, and IVB corresponded to stages of localized, regional, and distant metastasis, respectively, in the present study. Furthermore, we divided patients with cervical cancer into early-stage (CIS/localized) and advanced-stage (regional/distant metastasis) cancer groups.

For the diagnostic processes, we identified patients with cervical cancer who were diagnosed via cancer screening in the public and private sectors. The patients who were not diagnosed via cancer screening were designated as such. There were two kinds of such patients: (1) those who were diagnosed with cervical cancer during diagnoses/treatments for other diseases and (2) those who directly visited clinics/hospitals with some symptoms of ill health without undergoing cancer screening.

Furthermore, we classified patients into 10-year age categories (e.g., 20–29 years) considering the limited sample size. We also divided the patients into two groups—the younger (<50 years) and older (≥50 years) groups—based on the age of onset of cervical cancer decline [2].

### 2.3. Statistical Analysis

Using the 1985 national model population, we estimated the age-standardized and age-specific incidence rates of cervical cancer in women aged ≥20 years in Tochigi prefecture during the study period. The AAPC and 95% CI were calculated for the 5-year study period (2009–2013). Joinpoint regression analysis, which shows the temporal trend of incidence by estimating the percent change over time using piecewise log-linear regression, and the Joinpoint regression program (version 4.7.0.0) (National Cancer Institute, Bethesda, MD, USA) from the National Cancer Institute were applied according to the methodologies used in previous studies [8,9].

Moreover, to elucidate the trend of increasing cervical cancer incidence and to identify the population at risk, we estimated age-specific AAPCs, stratified by pathological subtypes. Additionally, we stratified the analysis according to the cancer stage and the diagnostic process. In this additional analysis, we used the binary age category (<50 years or ≥50 years), considering the limited sample size.

In a subgroup analysis, we used a limited study sample of 859 patients with invasive cancer (C53 in ICD-10), which corresponded to cervical cancer patients at FIGO stages IA1 and greater. We performed the same analytic procedure, although additional stratifications with cancer stage and diagnostic process were not possible due to the limited sample size. Alpha was set at 0.05, and all P-values were two-sided. For statistical analyses, data were analyzed using the Joinpoint regression program [17] and the IBM SPSS Statistics 25 version (IBM, Armonk, NY, USA) for Windows.

## 3. Results

The overall age-standardized incidence was 45.1/100,000 population during the 5-year study period; although the annual incidence rate did not significantly increase, it tended to show a potential increase (Table 1).

**Figure 1:**
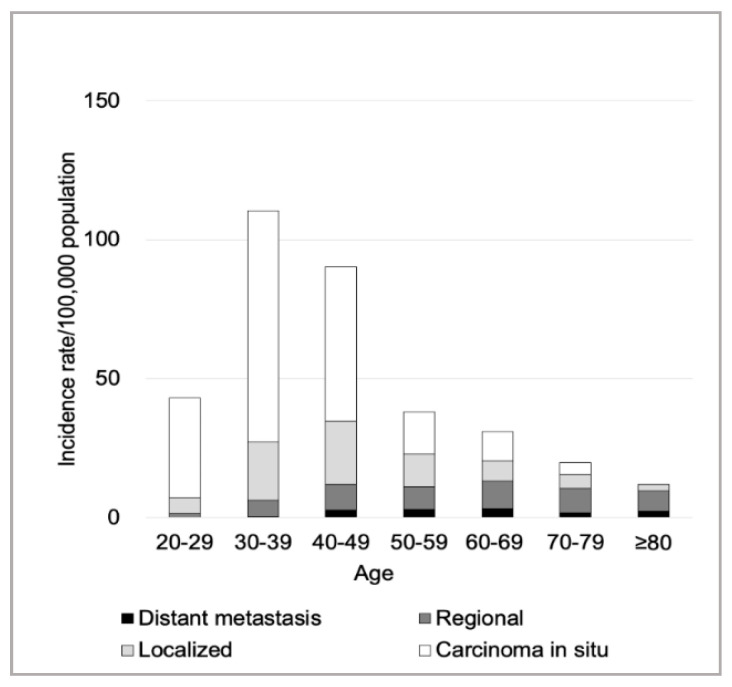
Age-specific cervical cancer incidence for each cancer stage between 2009 and 2013. The incidence of carcinoma in situ (CIS) and early-stage cancer was highest in women aged 30–39 years. The age-specific incidences of regional and distant metastatic cancers/100,000 population in their 40s, 50s, 60s, 70s, and ≥80s were, respectively, as follows: regional cancer, 9.2, 8.1, 10.0, 8.7, and 7.2; distant metastatic cancer, 2.9, 2.9, 3.2, 1.9, and 2.3.

However, the age-specific incidence was highest in women aged 30–39 years (Figure 1), and the incidence showed a significant increase in this age group: AAPC 20.0 (95% CI: 9.9–31.1; Table 1). In addition, the incidence of CIS, as well as early-stage cancer, was highest in women aged 30–39 years (Figure 1), and the incidence showed a significant increase (Table 1) (AAPCs for CIS and early-stage cancers: 17.8 (95% CI: 6.4–30.4) and 12.6 (95% CI: 2.7–23.4), respectively). The dominant histological subtype was SCC (88.2%), and the overall incidence of SCC showed a potential increase (Table 1). The percentage of patients diagnosed via cancer screening was 46.8% (Table 1). No joinpoints were observed in the joinpoint regression analysis (Figure S1).

**Figure 2:**
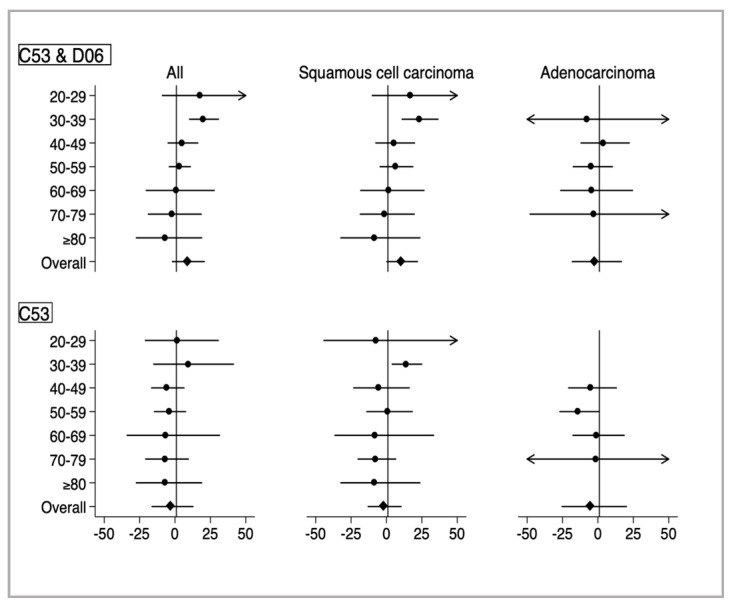
Age-specific average annual percent change for cervical cancer stratified by histological subtype. The average annual percent change (dot) and 95% confidence intervals (line) were estimated using joinpoint regression analyses. Upper panels indicate data of overall cervical cancer patients, including those with carcinoma in situ (ICD-10, D06) and invasive cancer (C53). Lower panels indicate data of those with only invasive cervical cancer (C53).

In the analyses stratified by histology (Figure 2 and Table 2), the increase in age-specific AAPC was only significant in SCC among women aged 30–39 years (AAPC 23.1, 95% CI: 10.7–36.8).

In the additional analyses stratified by cancer stage and diagnostic process, the incidence of early-stage cancer through the non-cancer screening processes, particularly that specific to the SCC subtype among younger women, showed a significant increase (Table 3).

In the subgroup analysis limited to invasive cervical cancer (C53 in ICD-10), the overall age-standardized incidence was 15.8/100,000 population (Table 1). The age-specific incidence stratified by histology showed a similar pattern (Figure 2 and Table 2).

## 4. Discussion

In this study, we showed that the incidence of cervical cancer increased among younger women in Japan. Specifically, women in their 30s are at risk for early-stage cervical cancer with the SCC subtype. Additionally, this trend has been increasingly observed in cancer patients not diagnosed via cancer screening processes.

Our findings confirm that the recent concerns for eliminating cervical cancer in Japan are valid—the incidence of cervical cancer is increasing, particularly among young women. In this study, the age-standardized incidence of overall cervical cancer in Japan was higher than that in Western countries [3], which is in concordance with the findings of previous studies [7,8,9]. Likewise, Yagi et al. reported a continuous increase in SCC cervical cancer (including that in corpus cancer) among younger women aged <40 years in Osaka prefecture since 2000 (AAPC 5.9%/year) [9]. Similarly, Utada et al. reported a skyrocketing of CIS cases among younger women aged 30–39 years in Nagasaki prefecture since 2007 (AAPC 19.0%/year) [8].

Although the reasons for this trend are yet to be fully determined, it might be related to the trajectory of sexual practice, including the prevalence of HPV infection and sexual behavior. In a hospital-based study in Japan, later birth cohorts had a higher prevalence of HPV infection, including HPV 16/18 [18]. This difference might be associated with early sexual debut and having multiple sexual experiences with different partners, as well as the less frequent use of condoms among recent birth cohorts [19]. Indeed, the increasing trend of cervical cancer incidence differed slightly across Japan (including in our study) [8,9], reflecting geographical differences in sexual practice. However, the cervical cancer incidence among younger South Korean or Japanese American women did not show an increase, implying that the increasing rates of cancer screening may offset even cultural shifts toward a higher risk for cervical cancer [9].

In this study, contrary to our expectations, diagnosis via the non-cancer screening process played a role in the increased detection of cervical cancer. We need to elucidate the mechanism further because early-stage cancer patients (particularly those with CIS) have few symptoms, and regular practice would merely detect this type of cancer. One potential explanation (yet to be determined due to our limited data) might include a routine Pap smear during regular pregnancy checkups or infertility treatments in Japan; these processes are not recognized as “cancer screening” in prevention strategies. The average age of women at first birth in Japan in 2017 was 30.7 years [20], and the birth rate among women aged 30–39 was the highest ever [21]. Besides, the number of married couples with a history of receiving infertility treatment has increased to 18.2% [22]. Nevertheless, the regular checkup is not designed to prevent cervical cancer in this high-risk population and does not cover women who are not willing to conceive. Therefore, it is necessary to increase the rate of cancer screening at the population level, particularly in high-risk younger women.

For older women, although the age-standardized incidence of advanced-stage cancer (i.e., life-threatening cancer) remained constant regardless of age, with a higher percentage of advanced-stage (Figure 1), the overall incidence of cervical cancer was low. In a British study, adequate screening by 50–65 years of age tended to reduce the risk of subsequent cervical cancer [23]. Cervical cancer screening should aim to decrease the overall mortality at the population level; however, healthcare resources are limited in Japan. Currently, as the cervical cancer screening program does not have an upper age limit (i.e., all women aged ≥20 are eligible), further studies addressing the age limit from the perspective of health economics are warranted.

This study has several limitations. First, the study period was short, and the data with the small sample size are only for a single prefecture in the metropolitan region (with an approximate population of only 1.5% of the Japanese population), thereby limiting the external generalizability. In addition, our staging was not entirely according to the FIGO classification, and potential misclassifications might have been introduced. However, we followed the latest diagnostic classification (the Bethesda system), and the age distribution of patients with cervical cancer (from the Tochigi prefecture) paralleled those reported in previous studies and national statistics [7,8,9]. Second, we could not explicitly specify the increasing trend in public cancer screening, and HPV-infection and smoking-related data were not available. However, in Japan, the cancer screening rate is low, and smoking is attributable to approximately 2% of the incidence of overall cervical cancer; further, the rate of smoking has been decreasing among women in Japan [9]. Third, due to the limitations of our dataset, we did not assess socioeconomic disparities [24]. As socially disadvantaged women are less likely to have access to cancer screening or HPV vaccination even under the universal health coverage system in Japan, future studies should address the socioeconomic gap affecting the increasing trend of cervical cancer.

Despite these limitations, using high-quality population-based data, we specifically identified the prioritized target population to prevent cervical cancer—the recent increase in cervical cancer in Japan is likely attributable to the early-stage, HPV-related cancer subtype among young women in their 30s who are unlikely to undergo cervical cancer screening. Although cancer prevention has to be a government policy, in a population poll conducted by the Cabinet Office in Japan, the reasons for individuals not undergoing cancer screening included “not having time to undergo cancer screening” (30.6%) and “no need due to their confidence in their health” (29.2%) [25]. This poll highlighted insufficient public education as the probable reason for the low cancer screening rate. Hence, public awareness that screening can eliminate the risk of cervical cancer at the population level, particularly in young women, should be promoted, while considering the introduction of the combination of HPV testing and Pap smear. Furthermore, the HPV vaccination program, which was suspended in 2013 in Japan, should be resumed, and cancer screening should be promoted to eliminate cervical cancer.

## 5. Conclusions

In Japan, the incidence of cervical cancer has been increasing in young women, with the trend being most pronounced in those with early-stage cancer of the SCC subtype diagnosed without cancer screening processes. The national prevention strategy should explicitly incorporate HPV vaccination and cancer screening for eliminating cervical cancer.

## Figures and Tables

**Table 1:** Characteristics of cervical cancer registry cases in the Tochigi prefecture (2009–2013).

Variables	2009	2010	2011	2012	2013	2009–2013	AAPC (95% CI)
	N (%)	N (%)	N (%)	N (%)	N (%)	N (%)	
Overall	331	409	456	398	516	2110	8.8 (−2.2, 21.1)
Age, years, mean (SD)	46.8 (15.04)	45.5 (14.77)	45.2 (14.40)	43.8 (13.94)	43.2 (13.82)	44.8 (14.39)	
Age category (years)							
20–29	29 (8.8)	39 (9.5)	47 (10.3)	30 (7.5)	60 (11.6)	205 (9.7)	17.6 (−9.3, 52.6)
30–39	90 (27.2)	132 (32.3)	143 (31.4)	153 (38.4)	193 (37.4)	711 (33.7)	20.0 (9.9, 31.1)
40–49	90 (27.2)	106 (25.9)	126 (27.6)	110 (27.6)	126 (24.4)	558 (26.4)	5.1 (−5.3, 16.5)
50–59	51 (15.4)	52 (12.7)	52 (11.4)	45 (11.3)	54 (10.5)	254 (12.0)	3.1 (−4.4, 11.2)
60–69	42 (12.7)	43 (10.5)	55 (12.1)	31 (7.8)	53 (10.3)	224 (10.6)	0.7 (−20.7, 28.1)
70–79	19 (5.7)	25 (6.1)	22 (4.8)	16 (4.0)	23 (4.5)	105 (5.0)	−2.0 (−19.2, 18.9)
≥80	10 (3.0)	12 (2.9)	11 (2.4)	13 (3.3)	7 (1.4)	53 (2.5)	−7.2 (−27.7, 19.2)
Historical subtype							
Squamous cell carcinoma	282 (85.2)	360 (88.0)	390 (85.5)	359 (90.2)	470 (91.1)	1861 (88.2)	10.5 (−0.1, 22.3)
Adenocarcinoma	40 (12.1)	41 (10.0)	49 (10.7)	31 (7.8)	40 (7.8)	201 (9.5)	−2.3 (−18.5, 17.0)
Other	9 (2.7)	8 (2.0)	17 (3.7)	8 (2.0)	6 (1.2)	48 (2.3)	−5.4 (−45.9, 65.4)
Stage							
Carcinoma in situ	158 (50.8)	231 (56.5)	253 (55.5)	261 (65.6)	348 (67.4)	1251 (59.3)	17.8 (6.4, 30.4)
Localized	92 (29.6)	88 (21.5)	114 (25.0)	83 (20.9)	94 (18.2)	471 (22.3)	−0.2 (−13.9, 15.6)
Regional	67 (21.5)	70 (17.1)	71 (15.6)	44 (11.1)	55 (10.7)	307 (14.5)	−7.7 (−21.9, 9.1)
Distant metastasis	14 (4.5)	20 (4.9)	18 (3.9)	10 (2.5)	19 (3.7)	81 (3.8)	0.6 (−24.9, 34.7)
Early-stage	250 (75.5)	319 (78)	367 (80.5)	344 (86.4)	442 (85.7)	1722 (81.6)	12.6 (2.7, 23.4)
Advanced-stage	81 (24.5)	90 (22.0)	89 (19.5)	54 (13.6)	74 (14.3)	388 (18.4)	−6.0 (−22.3, 13.6)
Diagnostic process							
Via cancer screening	149 (45.0)	203 (49.6)	206 (45.2)	176 (44.2)	253 (49.0)	987 (46.8)	9.6 (−5.7, 27.4)
Not via cancer screening	182 (55.0)	206 (50.4)	250 (54.8)	222 (55.8)	263 (51.0)	1123 (53.2)	8.1 (−1.0, 18.2)
Death certificate only, %	0.9	0.5	0.9	0.7	0.6	0.6	
Age-standardized incidence (C53 & D06) ^a^	33.9	42.9	48.2	43.6	57.7	45.1	
Age-standardized incidence (C53) ^a^	15.9	15.8	18.7	12.8	16.0	15.8	

Abbreviations: SD, standard deviation; AAPC, average annual percent change; CI, confidence interval; N, number. ^a^ Age-standardized incidence/100,000 population was calculated with the 1985 national model population.

**Table 2:** Age-specific average annual percent change estimated with joinpoint regression stratified by histological subtype for 2009–2013.

Age Category	N	AAPC (95% CI)
All		
20–29	205	17.6 (−9.3, 52.6)
30–39	711	20.0 (9.9, 31.1)
40–49	558	5.1 (−5.3, 16.5)
50–59	254	3.1 (−4.4, 11.2)
60–69	224	0.7 (−20.7, 28.1)
70–79	105	−2.0 (−19.2, 18.9)
≥80	53	−7.2 (−27.7, 19.2)
Squamous cell carcinoma		
20–29	194	17.0 (−10.4, 52.9)
30–39	651	23.1 (10.7, 36.8)
40–49	492	5.2 (−7.9, 20.2)
50–59	209	6.4 (−4.9, 19.0)
60–69	185	1.6 (−18.6, 26.9)
70–79	85	−1.4 (−18.9, 20.0)
≥80	45	−8.6 (−32.6, 24.0)
Adenocarcinoma		
20–29	10	Not available
30–39	47	−8.0 (−57.1, 97.7)
40–49	54	3.7 (−12.3, 22.5)
50–59	37	−4.7 (−17.8, 10.6)
60–69	32	−4.4 (−26.7, 24.8)
70–79	17	−2.9 (−48.3, 82.4)
≥80	4	Not available

Abbreviation: AAPC, average annual percent change; CI, confidence interval; N, number.

**Table 3:** Age-specific average annual percent change further stratified by cancer stage and screening process.

Variables	All			Squamous Cell Carcinoma			Adenocarcinoma		
	N	AAPC	95% CI	N	AAPC	95% CI	N	AAPC	95% CI
Early-stage cancer									
Via cancer screening									
Overall	924	11.6	−3.8, 29.5	853	12.5	−3.3, 31.0	59	6.8	−21.0, 44.3
Age <50	729	15.0	−3.9, 37.6	677	15.7	−3.5, 38.8	45	12.4	−27.0, 73.0
Age ≥50	195	3.3	−0.3, 7.0	176	4.4	−0.3, 9.2	14	0.4	−29.6, 43.2
Not-via cancer screening									
Overall	798	13.6	4.9, 23.1	712	15.7	6.9, 25.3	74	−2.1	−23.4, 25.2
Age <50	623	16.5	8.7, 25.0	571	18.0	10.6, 26.0	43	−0.1	−30.1, 42.7
Age ≥50	175	7.6	−11.1, 30.3	141	11.2	−10.5, 38.2	31	−3.1	−23.2, 22.1
Advanced-stage cancer									
Via cancer screening									
Overall	63	−16.5	−34.0, 5.6	49	−14.8	−27.9, 0.6	11	−23.7	−48.4, 12.8
Age <50	26	−2.0	−27.1, 31.9	21	4.3	−26.5, 48.1	4	n/a	
Age ≥50	37	−26.2	−44.5, −1.9	28	−27.9	−48.6, 1.1	7	−20.2	−35.3, −1.6
Not-via cancer screening									
Overall	325	−3.9	−20.0, 15.5	247	−3.5	−22.2, 19.6	57	−6.3	−27.7, 21.3
Age <50	96	−2.9	−23.1, 22.7	68	−1.6	−29.9, 38.0	19	−2.8	−47.1, 78.9
Age ≥50	229	−4.6	−20.4, 14.4	179	−4.3	−20.6, 15.5	38	−3.6	−34.2, 41.3

Abbreviation: AAPC, average annual percent change; CI, confidence interval; N, number.

## Supplementary Materials

The following are available online at https://www.mdpi.com/1660-4601/17/20/7401/s1, Figure S1: Age-specific average annual percent change for cervical cancer incidence in Tochigi prefecture (2009–2013).
