# Supplementary material for: Recent Increasing Incidence of Early-Stage Cervical Cancers of the Squamous Cell Carcinoma Subtype among Young Women
Source: Int J Environ Res Public Health. 2020 Oct 12;17(20):7401. doi: 10.3390/ijerph17207401 (PMC7599510; doi:10.3390/ijerph17207401)
Supplement: Supplementary file 1 [file ijerph-17-07401-s001.pdf]

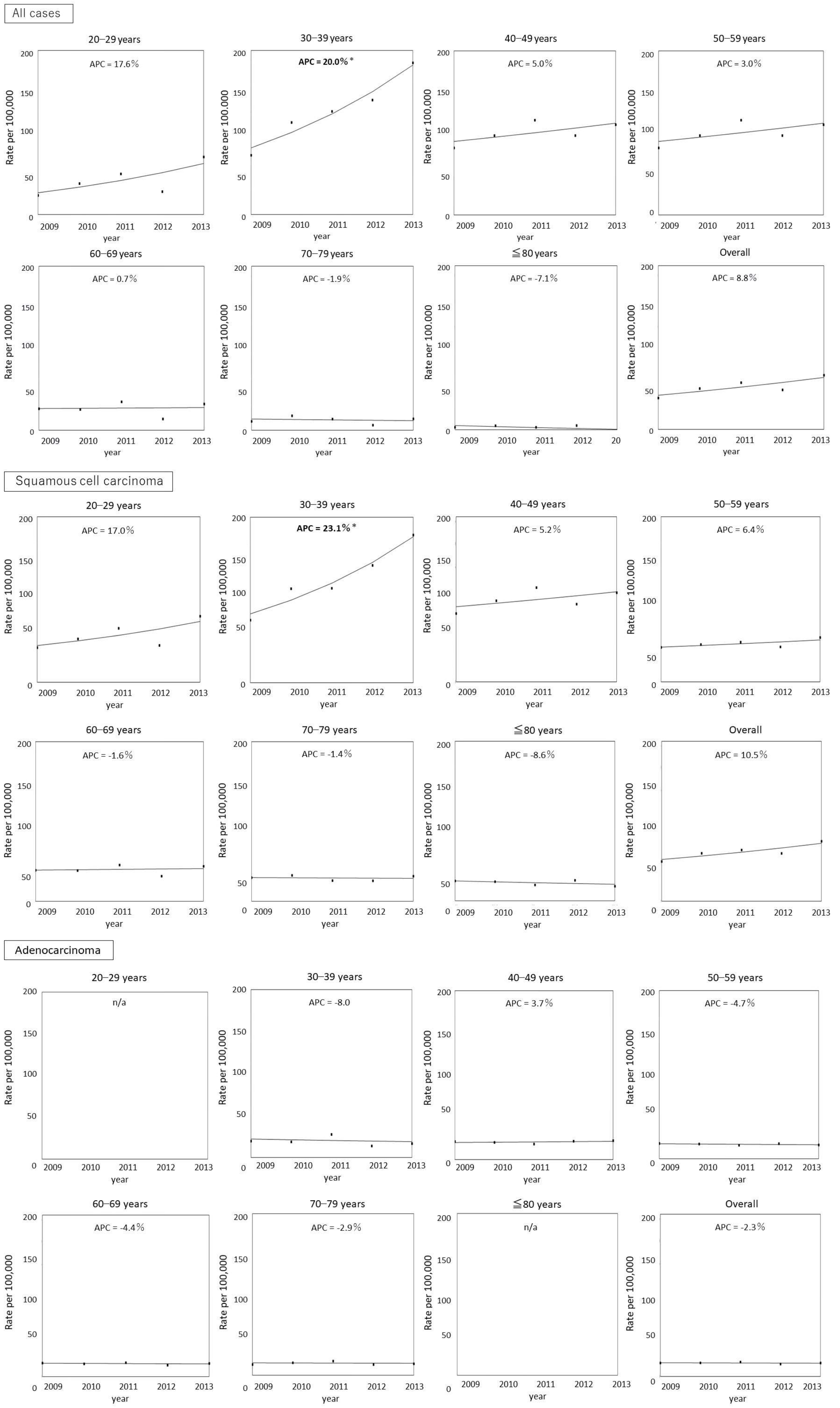

Figure S1. Age-specific average annual percent change for cervical cancer incidence in Tochigi prefecture (2009–2013). These figures were generated with the Joinpoint Regression Program (version 4.7.0.0) from the National Cancer Institute.
